# Lower dose and weekly schedules of selinexor in multiple myeloma - updated evidence on safety and efficacy

**DOI:** 10.3389/fonc.2025.1649493

**Published:** 2025-09-22

**Authors:** Muhamed Baljevic, Gary Schiller, Tomer M. Mark, Dane R. Van Domelen, Cristina Gasparetto

**Affiliations:** ^1^ Vanderbilt-Ingram Cancer Center, Vanderbilt University Medical Center, Nashville, TN, United States; ^2^ David Geffen School of Medicine at University of California, Los Angeles (UCLA), Los Angeles, CA, United States; ^3^ Karyopharm Therapeutics Inc., Newton, MA, United States; ^4^ Division of Hematologic Malignancies and Cellular Therapy, Department of Medicine, Duke University Medical Center, Durham, NC, United States

**Keywords:** selinexor, multiple myeloma, drug administration schedules, exportin 1, antagonists, inhibitors, combination drug therapy, clinical efficacy

## Abstract

**Background:**

Selinexor, a first-in-class, oral exportin-1 inhibitor, showed activity in penta-refractory multiple myeloma (MM) in early trial exploration; however, the side-effect profile of twice-weekly dosing led to hesitant incorporation into widespread practice. Here, our objective is to provide updated clinical evidence highlighting the preserved efficacy and improved tolerability of once-weekly selinexor at lower doses in patients with previously treated MM compared to twice-weekly regimens.

**Methods:**

Patient-level data from the BOSTON, STOMP, STORM, and XPORT-MM-028 clinical trials were systematically evaluated to elucidate relationships between selinexor dosing schedule, regimen toxicities, and efficacy in patients with MM that had progressed after at least one prior therapy.

**Results:**

Updated results on once-weekly selinexor in combination with other anti-MM agents showed a reduced adverse event profile and improved tolerability compared with twice-weekly selinexor regimens, without compromise in efficacy. Furthermore, new data from several regimens with weekly selinexor delivery suggest that patients who had selinexor dose reductions or were treated in cohorts with a lower selinexor starting dose had reduced rates of adverse events, and superior durations of response. Weekly selinexor in combination with pomalidomide or carfilzomib in particular showed efficacy in difficult-to-treat, multiclass relapsed/refractory MM, including MM refractory to prior BCMA-directed therapies.

**Conclusions:**

In a rapidly evolving field of previously treated MM, lowering of selinexor dose and frequency into weekly regimens showed a more feasible and tolerable treatment with continued efficacy when compared to twice-weekly schedules, paving the path for effective management of multiclass refractory MM, including patients with very advanced disease.

## 1 Introduction

**Figure 1:**
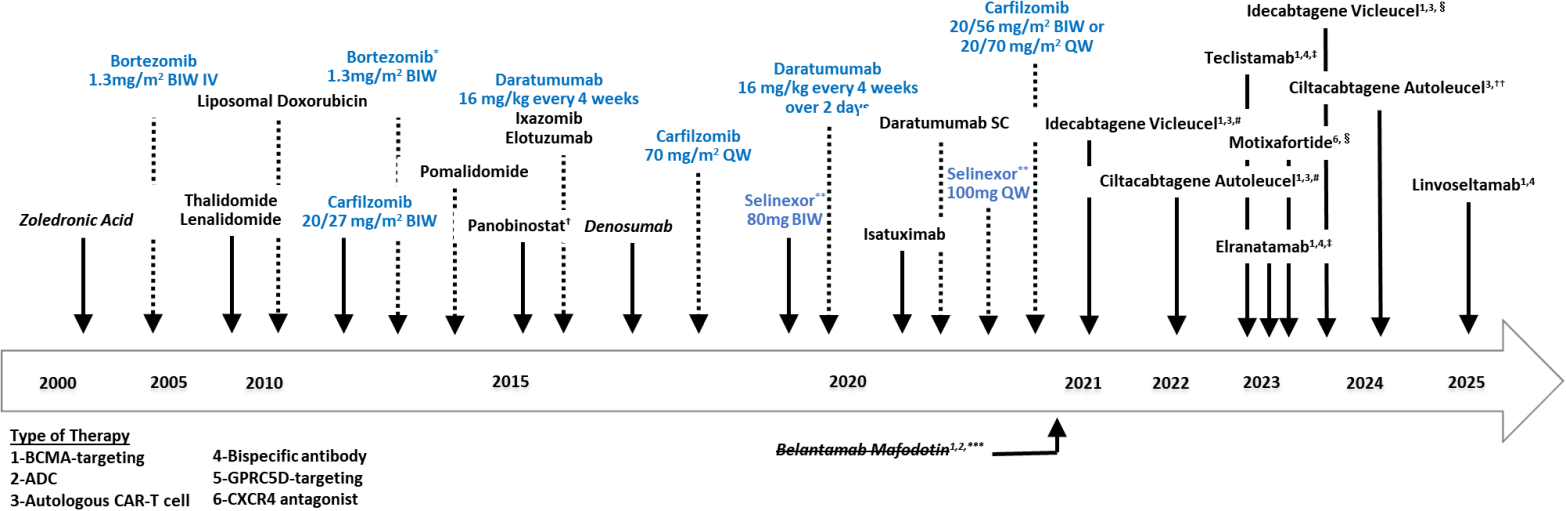
Two decades of multiple myeloma regulatory approvals. ADC, antibody dependent cytotoxicity; BCMA, B cell maturation antigen; BIW, twice-weekly; CAR-T, chimeric antigen receptor T-cell; CXCR4, C-X-C chemokine receptor type 4; GPRC5D, G protein-coupled receptor family C group 5 member D; SC, subcutaneous; QW, once-weekly. Drugs in blue have additional changes in doses/route of administration following drug approval. *Bortezomib dose remained the same and route of administration was updated to include sub-cutaneous in addition to intravenous. **Accelerated approval granted by the FDA in July 2019; approved in December 2020 in combination with bortezomib and dexamethasone; granted full approval by the European Medicines Agency (EMA) in May 2022. ***Accelerated approval granted by the FDA, withdrawn in November 2022 after negative results in confirmatory trial (DREAMM-3). ^†^Approval was granted by the FDA in Nov 2023 for patients with relapsed or refractory multiple myeloma (RRMM) who have received at least 4 prior lines of therapy. ^‡^Conditional marketing authorization granted by the EMA and granted accelerated approval by the FDA. ^#^Approval granted by the US FDA and conditional marketing authorization granted by the EMA. ^§^Approval granted to include sooner after two or more lines of therapy. Originally approved for later line use ≥4 prior lines of therapy. ^††^Approval granted to include use at first relapse in adult patients refractory to lenalidomide after 1–3 prior lines. Originally approved for later line use ≥4 prior lines of therapy.

Multiple myeloma (MM) treatment is ever-evolving (**Figure 1**). Prior to recent advent of chimeric antigen receptor (CAR) T cell and bispecific antibodies, the single-agent overall response rates (ORRs) in previously treated MM ranged from 20%-30% (**Table 1**), highlighting the need to discover and combine new mechanisms of action to achieve optimal clinical responses. In 2019, selinexor (S), the first-in-class, oral XPO - 1 inhibitor, was approved in combination with dexamethasone for the treatment of heavily pre-treated relapsed/refractory myeloma. Overexpression of XPO - 1 is correlated with poor prognosis in a number of malignancies, including MM (9, 10, 12–20). Selectively targeting XPO - 1 interferes with nucleocytoplasmic transport and reactivates tumor suppressor proteins, while also blocking translation of oncogene-coding mRNAs (12). The Phase 2b Selinexor in the Treatment of Relapsed Myeloma (STORM) study (NCT02336815) evaluating the doublet combination of selinexor 80 mg plus dexamethasone 20 mg (Sd) twice-weekly showed an ORR of 26%, a median time to first response of 4.1 weeks, and a median progression free survival (PFS) of 3.7 months (9, 10, 12). Sd also showed improved overall survival (OS) in matched cohorts in community and academic settings. Yet, although it was a new option for penta-refractory MM, selinexor incorporation in everyday practice was hindered by the side effect profile of this twice-weekly combination.

**Table 1:** Previous single agent (or dexamethasone doublet) regulatory landmarks in RRMM.

Agent	Setting	Prior Lines of Treatment (median)	Triple Class RRMM (%)	Single Agent ORR	Dex Doublet ORR	EFS/PFS/TTP
Thalidomide (1)	Relapsed MM Post-ASCT	–	0%	25%	46%	EFS 20% at 2 years
Bortezomib (2)	RRMM: Steroids 99.5%, Thal 83%	6	0%	35%	–	TTP 6.6 months
Lenalidomide (3–5)	Thal 36 - 80% V 8 - 21%	–	0%	25%	61%	TTP 13.4 months (Rd)
Carfilzomib (6)	RRMM: V/IMiD ~100%	5	0%	24%	–	PFS 3.7 months
Pomalidomide (7)	RRMM: V/R 100%	5	0%	18%	33%	PFS 2.7 months PFS 4.2 months (Pd)
Daratumumab (8)	RRMM: V/R 98 - 99%	5	0%	31%	–	PFS 4.0 months
Selinexor (9)	RRMM: 100% penta-exposed, triple-class refractory MM	7	100%	–	26%	PFS 3.7 months
Selinexor (10)	100% penta-refractory MM	8	100%	–	25%	PFS 2.8 months
Belantamab Mafodotin (11)	RRMM	7	100%	32%	–	PFS 2.8 months^*^

ASCT, autologous stem cell transplant; BCMA, B-cell maturation antigen; Dex, dexamethasone; EFS, event-free survival; IMiD, immunomodulatory drug; MM, multiple myeloma; ORR, overall response rate; Penta, disease refractory to at least 2 proteasome inhibitors, at least 2 IMiDs, and anti-CD38 mAb; Pd, pomalidomide dexamethasone; PFS, median progression-free survival; Rd, lenalidomide dexamethasone; RRMM, relapsed and/or refractory multiple myeloma; Thal, thalidomide; TTP, time to progression; Triple, disease refractory to at least 1 proteasome inhibitor, 1 IMiD and 1 anti-CD38 monoclonal antibody (mAb); V, bortezomib.

^*^PFS was calculated after longer term follow-up of 13 months in the DREAMM - 2 study in patients who received belantamab mafodotin 2.5 mg/kg.

Here, we review the clinical efficacy and tolerability of selinexor in combination with various partner agents, with a focus on weekly versus twice-weekly dosing.

## 2 Material and methods

Patients with RRMM treated with selinexor were evaluated across multiple trials, including BOSTON (S-bortezomib, dexamethasone [SVd]) (12), Selinexor and Backbone Treatments of Multiple Myeloma Patients (STOMP) (NCT02343042) (S-pomalidomide, dexamethasone [SPd] (16), S-lenalidomide, dexamethasone [SRd] (13, 14), S-daratumumab, dexamethasone [SDd] (15), and S-carfilzomib, dexamethasone [SKd] (21) arms), STORM (9), and XPORT-MM-028 (NCT04414475) (SPd arm). These regimens incorporated both weekly and twice-weekly selinexor dosing schedules (**Table 2**). The impacts of selinexor posology on regimen toxicities, treatment duration, need for dose modifications, and efficacy in RRMM were examined.

**Table 2:** Efficacy and safety of selinexor regimens.

	STORM[9] (Sd)* (n=123)			BOSTON[12] (SVd) (n=195)			SPd-60[16] (n=20)			SPd-40[16] (n=27)			SRd[16] (n=24)**		
Selinexor dose, mg	80 mg BIW			100 mg QW			60 mg QW			40 mg QW			60 mg QW^**^		
Efficacy															
Rx lines, median (range)	7 (3 - 18)			1 (1 - 3)			3 (1 - 9)			2 (1 - 5)			1 (1 – 8)		
ORR, n (%)	32 (26)			149 (76)			13 (65)			14 (50)			12 (60.0)		
mPFS, months (95% CI)	3.7 (3.0 - 5.3)			13.9 (11.7-NR)			9.5 (7.6-NR)			18.4 (6.5-NR)			9.6 (5.6-NR)		
mOS, months (95% CI)	8.6 (6.2 - 11.3)			NR (NR-NR)			NR (9.3-NR)			NR (12.9-NR)			NR (NR-NR)		
mDOR, months (95% CI)	4.4 (3.7 - 10.8)			20.3 (12.5-NR)			8.6 (3.9-NR)			NR (17.5-NR)			NR (8.7-NR)		
Safety															
AEs, n (%)	Any	≥G3	G5	Any	≥G3	G5	Any	≥G3	G5	Any	≥G3	G5	Any	≥G3	G5
	123 (100)	117 (95)	12 (10)	194 (99)	166 (85)	12 (6)	20 (100)	19 (95)	0 (0)	27 (100)	25 (86)	0 (0)	24 (100)	23 (96)	4 (17)
Hematological TEAEs, n (%)															
Anemia	83 (68)	54 (44)	0 (0)	71 (36)	31 (16)	0 (0)	13 (65)	5 (25)	0 (0)	13 (46)	6 (21)	1 (4)	11 (46)	7 (29)	0 (0)
Neutropenia	49 (40)	26 (21)	0 (0)	29 (15)	17 (9)	0 (0)	15 (75)	12 (60)	0 (0)	18 (64)	18 (64)	0 (0)	15 (63)	15 (63)	0 (0)
Thrombocytopenia	90 (73)	72 (59)	0 (0)	117 (60)	77 (40)	0 (0)	9 (45)	5 (25)	0 (0)	12 (43)	7 (25)	0 (0)	18 (75)	16 (67)	0 (0)
Non-Hematological TEAEs, n (%)															
Decreased appetite	69 (56)	6 (5)	0 (0)	69 (35)	7 (4)	0 (0)	6 (30)	0 (0)	0 (0)	5 (18)	0 (0)	0 (0)	13 (54)	2 (8)	0 (0)
Decreased weight	62 (50)	1 (1)	0 (0)	51 (26)	4 (2)	0 (0)	5 (25)	0 (0)	0 (0)	6 (21)	0 (0)	0 (0)	11 (46)	2 (8)	0 (0)
Diarrhea	56 (46)	9 (7)	0 (0)	63 (32)	12 (6)	0 (0)	7 (35)	0 (0)	0 (0)	8 (29)	^****^	0 (0)	12 (50)	0 (0)	0 (0)
Fatigue	78^ǁ^ (63)	25^ǁ^ (20)	0 (0)	82 (42)	26 (13)	0 (0)	15 (75)	3 (15)	0 (0)	13 (47)	1 (4)	0 (0)	13 (54)	4 (17)	0 (0)
Nausea	88 (72)	12 (10)	0 (0)	98 (50)	15 (8)	0 (0)	14 (70)	0 (0)	0 (0)	9 (32)	2 (7)	0 (0)	16 (67)	1 (4)	0 (0)
Peripheral neuropathy	8 (7)	2 (2)	0 (0)	63 (32)	9 (5)	0 (0)	2 (10)	0 (0)	0 (0)	3 (11)	0 (0)	0 (0)	2 (8)	0 (0)	0 (0)
Vomiting	47 (38)	4 (3)	0 (0)	40 (21)	8 (4)	0 (0)	5 (25)	0 (0)	0 (0)	4 (13)	0 (0)	0 (0)	9 (38)	0 (0)	0 (0)
TEAEs leading to dose modification^ǁ^	98 (80)^±^			173 (89)			15 (75)			19 (68)			30 (94)		
TEAEs leading to dose reduction^ǁ^	69 (56)			141 (72)			10 (50)			9 (32)			22 (69)		
TEAEs leading to dose discontinuation^ǁ^	40 (33)			41 (21)			2 (10)			5 (18)			3 (9)		
	SDd[15] (n=34)***			SKd[21] (n=32)			Anti-CD38 RRMM[22] (n=62)^††^						Anti-BCMA RRMM[23] (n=7)		
Selinexor dose, mg	100 mg QW^†^			80 mg QW^‡^			40 mg or 100 mg QW; 60 mg or 80 mg BIW or QW						40 mg, 60 mg, 80 mg, or 100 mg QW		
Efficacy															
Rx lines, median (range)	3 (2 - 10)			4 (1 – 8)			4 (1 – 11)						6 (4 – 10)		
ORR, n (%)	22 (69)			25 (78)			58 (36)						4 (57.1)		
mPFS, months (95% CI)	12.5 (7.6-NR)			15.0 (12.0-NR)			10.9 (7.6-NR)						6.0 (5.9-NR)		
mOS, months (95% CI)	NR (17.3-NR)			NR (NR-NR)			20.4 (15.2-NR)						14.8 (9.6-NR)		
mDOR, months (95% CI)	11.4 (9.7-NE)			22.7 (11.8-NR)			13.1 (11.5-NR)						NR (3.7-NR)		
Safety															
AEs, n (%)	Any	≥G3	G5	Any	≥G3	G5	Any		≥G3		G5		Any	≥G3	G5
	34 (100)	31 (91)	0 (0)	32 (100)	24 (75)	1 (3)	61 (98)		50 (81)		1 (2)		7 (100)	7 (100)	0 (0)
Hematological TEAEs, n (%)															
Anemia	24 (71)	12 35)	0 (0)	17 (53)	6 (19)	0 (0)	32 (52)		17 (27)		0 (0)		5 (71)	3 (43)	0 (0)
Neutropenia	17 (50)	9 (26)	0 (0)	10 (31)	2 (6)	0 (0)	24 (39)		14 (23)		0 (0)		3 (43)	3 (43)	0 (0)
Thrombocytopenia	24 (71)	16 (47)	0 (0)	23 (72)	15 (47)	0 (0)	35 (57)		22 (36)		0 (0)		5 (71)	5 (71)	0 (0)
Non-Hematological TEAEs, n (%)															
Decreased appetite	16 (47)	1 (3)	0 (0)	17 (53)	1 (3)	0 (0)	27 (44)		2 (3)		0 (0)		2 (29)	0 (0)	0 (0)
Decreased weight	10 (29)	1 (3)	0 (0)	13 (41)	0 (0)	0 (0)	23 (37)		0 (0)		0 (0)		3 (43)	0 (0)	0 (0)
Diarrhea	18 (53)	3 (10)	0 (0)	12 (38)	0 (0)	0 (0)	28 (45)		3 (5)		0 (0)		3 (43)	0 (0)	0 (0)
Fatigue	24 (71)	6 (18)	0 (0)	17 (53)	3 (9)	0 (0)	32 (52)		4 (7)		0 (0)		3 (43)	0 (0)	0 (0)
Nausea	25 (74)	3 (10	0 (0)	23 (72)	2 (6)	0 (0)	46 (74)		3 (5)		0 (0)		6 (86)	0 (0)	0 (0)
Peripheral neuropathy	2 (6)	0 (0)	0 (0)	7 (22)	1 (3)	0 (0)	8 (13)		2 (3)		0 (0)		0 (0)	0 (0)	0 (0)
Vomiting	11 (32)	1 (3)	0 (0)	6 (19)	1 (3)	0 (0)	19 (31)		2 (3)		0 (0)		1 (14)	0 (0)	0 (0)
TEAEs leading to Dose modification^ǁ^	--^‡‡^			26 (81)			50 (81)						--^‡‡^		
TEAEs leading to Dose reduction^ǁ^	22 (65)			22 (69)			38 (61)						--^‡‡^		
TEAEs leading to Dose discontinuation^ǁ^	5 (15)			5 (16)			8 (13)						--^‡‡^		

TEAE, treatment-emergent adverse event; BCMA, B-cell maturation antigen; BIW, twice-weekly; CBR, clinical benefit rate; CI, confidence interval; CR, complete response; D, dara-tumumab; d, dexamethasone; G, grade; K, carfilzomib; mDOR, median duration of response; mOS, median overall survival; mPFS, median progression-free survival; MR, minimal response; NR, not reached; ORR, overall response rate; P, pomalidomide; PD, progressive disease; PR, partial response; QW, once-weekly; R, lenalidomide; RRMM, relapsed/refractory multiple myeloma; SD, stable disease; V, bortezomib; VGPR, very good partial response; S, selinexor.

*Efficacy is based on modified intention-to-treat (mITT) population (n=122), safety based on safety population (n=123). One patient did not receive prior carfilzomib and was excluded from the intention-to-treat (ITT) population.

**Efficacy evaluable n=20; n=24 includes 12 patients at the RP2D 60 mg QW, 7 patients at 80 mg QW, and 5 patients at 60 mg BIW.

***Efficacy evaluable n=32.

****One TEAE in the SPd-40 group was missing grade ≥3 diarrhea.

^†^Other doses included 60 mg BIW.

^‡^Other doses include 60 mg and 100 mg QW.

^††^SPd (n=23), SVd (n=16), and SKd (n=23); 6 of 23 SPd, 10 of 23 SKd, and 16 of 16 SVd patients were treated at the listed RP2D doses. Additional patients were treated at different doses as part of the phase 1 portion of the STOMP Study.

^‡‡^Not listed.

^ǁ^Data on file. Data for fatigue as published in Chari et al., 2019[4] was reported as combination of fatigue and asthenia.

± Not including dexamethasone dose modifications.

## 3 Results

### 3.1 Lower selinexor dosing is tolerable and active

Despite the favorable response rates and clinical benefits seen in the STORM study, the 160 mg selinexor cumulative weekly dose resulted in high rates of adverse events (AEs) (**Table 2**) (17). The major Grade ≥3 non-hematological toxicities reported in STORM (n=202) were fatigue (22%) and nausea (9%). Tolerability, as assessed indirectly via median duration of exposure (mDOE), was 3.8 months (9, 10). Toxicities resulted in dose reductions and modifications in 80% of patients (9).

Subsequent studies evaluated lower selinexor doses and frequencies. The phase 3 BOSTON study (NCT 031110562) compared the efficacy of once-weekly (QW) selinexor 100 mg and subcutaneous bortezomib 1.3 mg/m^2^ combined with twice-weekly (BIW) dexamethasone 20 mg (SVd) to standard twice-weekly bortezomib and dexamethasone (Vd) in patients with MM that had progressive disease despite 1 – 3 prior lines of therapy (12). SVd received United States Food and Drug Administration (US FDA) approval in 2020 based on significantly improved PFS as determined by an independent review committee. After median follow-up of 13.2 months (SVd) and 16.5 months (Vd), median PFS was 13.93 and 9.46 months, respectively (HR 0.70 [95% CI 0.53 – 0.93], p=0.0075). The prespecified endpoint of ORR was 76.4% (SVd) versus 62.3% (Vd) (p = 0.0012), and ≥ very good partial response (VGPR) rates were 44.6% (SVd) versus 32.4% (Vd) (p=0.0082). The European Medicines Agency approved SVd in March 2021 for patients with MM who received at least one prior therapy.

Compared to twice-weekly 80 mg selinexor (Sd) in the STORM trial, the once-weekly 100 mg selinexor BOSTON regimen (SVd) showed improved tolerability, with ≥ Grade 3 non-hematologic toxicities of fatigue and nausea reduced to 13% and 8%, respectively, in the BOSTON study (9, 12). Selinexor dose reductions occurred in 65% of SVd-treated patients, with a median final dose of 71.4 mg/week. Patients who had a dose reduction compared to those before dose reduction had lower duration-adjusted AE incidence rates (18). Notably, patients in the BOSTON study who had a selinexor dose reduction had a longer median PFS compared to those who did not (16.6 versus 9.2 months), suggesting that lower selinexor doses have continued activity in RRMM (18). Furthermore, SVd treatment in the BOSTON study showed that ORR was higher among patients with dose reductions compared to those without (82% versus 67%). While some caveats to the interpretation of these results can be postulated, such as more motivation to maintain a regimen with dose reduction in patients who were already responding well, or the fact that patients with stable or slowly responding disease may have had earlier discontinuation of treatment, these data showed an emerging impact of lower dose, weekly schedules of selinexor in previously treated MM (18).

Following BOSTON, the STOMP trial, a multi-arm, open-label, Phase 1b/2 study, evaluated selinexor in various triplet and quadruplet combinations in newly diagnosed and relapsed/refractory MM. S-containing triplets comprised other commonly used anti-myeloma agents such as daratumumab (D) (15), pomalidomide (P) (19), lenalidomide (R) (13), and carfilzomib (K) (20), as outlined in **Table 2** (13, 15, 19, 20).

Among patients treated with SPd in STOMP (QW selinexor 40 mg [SPd-40] or 60 mg [SPd-60]) and XPORT-MM-028 (SPd-40), ORR and ≥VGPR in the SPd-40 cohort were 50% and 29%, respectively, and 65% and 30% in the SPd-60 cohort. Despite deeper responses in the SPd-60 cohort, as of June 30, 2023, mPFS was numerically longer, however did not reach statistical significance with SPd-40 (18.4 months [95% CI: 6.5, NR]) compared to SPd-60 (9.5 months [95% CI: 7.6, NR]) (16). The mDOE was also longer with SPd-40 (28 weeks) compared to SPd-60 (22 weeks). Similar to the BOSTON experience, lower selinexor dosing led to lower AE incidence, which was observed in the context of longer mDOE and mPFS for patients that were able to stay on treatment more reliably. Patients treated with SRd in STOMP (selinexor doses of 60 mg QW, 80 mg QW, and 60 mg BIW) had an ORR of 60%, PFS of 9.6 months, and OS that was not reached (**Table 2**), again showing activity for lower dose weekly selinexor. Overall, the total weekly selinexor dose in STORM was 1.6x higher than in BOSTON and 1.6 - 4x higher than preferred dosing in combination with other agents tested in STOMP cohorts. Taken together, lower selinexor doses, whether initiated at baseline or following dose modification, allows patients to stay on selinexor longer, thereby improving outcomes.

### 3.2 Weekly selinexor regimens offer value post-anti-CD38 mAb treatment

Most randomized trials evaluating second-generation proteasome inhibitors (PIs), immunomodulatory drugs (IMiDs), or agents with novel mechanisms of actions after early relapse have not included many patients with triple-class refractory (TCR) MM, largely due to trial-specific eligibility criteria (19, 20). However, with increasing use of frontline “quad” regimens, there is a growing need to address both non-responders and first-relapse patients with TCR MM. Recent studies in patients with RRMM treated with fewer prior lines of therapy have shown poor efficacy for triple-class exposed (TCE) or TCR MM treated with physician’s choice of subsequent therapy (24). Data from the LocoMMotion, MAMMOTH, and control arm of the KarMMa-3 trials, report that patients with at least TCE RRMM had PFS of 4.6, 3.4, and 4.4 months, respectively, and ORRs for the next line of treatment of 30 - 40% after anti-CD38 failure (24). These next line of treatments included different PIs, IMiDs, monoclonal antibodies, and alkylating chemotherapy regimens; selinexor-based combinations were used in < 1% of patients and therefore were not evaluated in the TCR MM context.

A retrospective analysis of 62 patients with RRMM from BOSTON and STOMP who had prior anti-CD38 exposure (median 4 prior lines), had ORRs of 52% (SPd), 56% (SVd), and 65% (SKd); only 6% of these patients received twice-weekly SPd dosing (22). Most patients were TCE and > 50% were TCR. Median PFS was 8.7 (SPd), 6.7 (SVd), and 15.0 (SKd) months and median OS was 20.4 months, with SPd at 9.6 months, SVd at 16.9 months, and SKd at 33.0 months (22). Adverse events for S-containing triplets were manageable with standard supportive care and dose modifications (**Table 2**). Grade ≥3 fatigue and nausea occurred in <5% of patients with SPd and SKd receiving with once-weekly dosing. Given historically poor outcomes in TCE RRMM with PIs, IMiDs, and anti-CD38 monoclonal antibodies who undergo drug-class recycling or turn to alkylator-based chemotherapy, these data suggest that once-weekly, appropriately selected S-based triplets offer a viable alternative, especially when access to T-cell-based immunotherapies is limited (22).

### 3.3 Selinexor in the new age of T-cell redirecting therapies

Chimeric antigen receptor T cell (CAR-T) and T-cell engaging bispecific antibody therapies have revolutionized our ability to manage RRMM. The single-agent activity of T-cell redirecting therapies established a new benchmark in RRMM, achieving ORR of 80 - 90% with CAR-T, and 50 - 70% with bispecific antibodies (**Table 3**). The two approved CAR-T cell products for MM, idecabtagene vicleucel and ciltacabtagene autoleucel, can be accessed in early lines of therapy based on the results of the KARMMA - 3 and the CARTITUDE - 4 phase 3 trials (39), while ongoing studies are generating data in the first line as well (40). This new era of MM therapeutics is welcome, however given that they are not curative, two issues must be addressed: 1) maximizing T-cell based therapy outcomes given their associated costs, logistical planning, and potential adverse effects; and 2) effectively addressing and treating a new population of RRMM that has failed bispecific antibody or CAR-T treatment. Early investigation into CAR-T efficacy has shown that factors such as T-cell exhaustion, an acquired inability for cytotoxic T-cells to target and eliminate tumors, may play a role in outcomes. A search for T-cell sparing agents to use in earlier line MM treatment to limit T-cell exhaustion and to support the efficacy of subsequent T-cell therapies is underway. IMiDs and cereblon E3 ligase modulatory drugs (CelMODs) have already been shown to increase cytotoxic T-cell activity as a downstream effect of cereblon targeting (41). Clinical data support the use of XPO - 1 inhibitors, such as low dose weekly selinexor, as potentially T-cell sparing in both the pre-apheresis stage (42) and the bridging period between apheresis and CAR-T infusion (43). Additional studies are underway to further investigate the interaction between XPO1 inhibitors, T cells, and the immune microenvironment. A retrospective study performed by the Myeloma CAR-T consortium examined the impact of various bridging regimens, as compared to no bridging, in patients with RRMM who received idecabtagene vicleucel. As reported, patients without the need for a bridge regimen to control the RRMM had the best outcomes, likely due to having more indolent disease. Amongst the patients who required bridging, those who received IMiD or S-based bridging had numerically longer PFS after CAR-T than those who received PI or alkylating chemotherapy bridging, with IMiD ± mAb combos showed comparable PFS to no-bridging (median PFS: 12.01 months vs. 11.48 months). In comparison to the IMiD ± mAb combination PFS, selinexor was 9.77 months, versus lower results with PI combos (6.41 months), and alkylator therapy (6.51 months) (43). These results suggest that S-based bridging combinations do not lead to inferior idecabtagene vicleucel outcomes, although the data specifying which doses and schedules of selinexor that were used as bridging was not reported.

**Table 3:** CAR-T cell and bispecific antibody single agent activity in RRMM.

Agent Class, Name		Target	Prior Treatments, median (range)	Single Agent ORR	PFS/DOR	Label Status
*CAR-T Cells*	Idecabtagene Vicleucel (25)	BCMA	6 (3 - 16)	82%	PFS 8.6 months at 24.8 months	FDA/EMA approved
	Ciltacabtagene Autoleucel (26, 27)	BCMA	6 (3 - 18)	98%	PFS 34.9 months DOR 33.9 months	
	P-BCMA-101 (28)	BCMA	7 (3 - 18)	67%	–^*^	FDA granted orphan drug designation
*Bispecific Antibodies*	Teclistamab-cqyv (29)	BCMA	5 (2 - 14)	63%	PFS 11.3 months DOR 18.4 months at 14.1 months	EMA approved FDA accelerated approval
	Elranatamab-bcmm (30)	BCMA	8^*^	70%	DOR NR at 6.3 months	
	Talquetamab-tgvs (31)	GPRC5D	6 (2 - 14)	70% at 405 µg/kg	–^*^	
	Cevostamab (32)	FcHR5	6 (2 - 15)	52%	–^*^	
	REG-5458 (33)	BCMA	5 (2 - 17)	63%	–^*^	
	ABBV-383 (34)	BCMA	5 (3 - 15)	68% at ≥ 40 mg	NR at 10.8 months	
	Zevorcabtagene autoleucel (35)	BCMA x CD3	4 (3 - 15)	93%	NR at 9 months	Under clinical development
	CART-ddbcma (36)	BCMA x CD3	–^*^	100%	–^**^	
	Linvoseltamab-gcpt (37)	BCMA x CD3	5 (2 - 16)	71%*	PFS NR DOR 29.4 months	FDA accelerated approval
	PHE885 (38)	BCMA	4 (2 - 10)	98%	––^*^	

CD, cluster of differentiation; CAR; chimeric antigen receptor; RRMM, relapsed and/or refractory multiple myeloma; BCMA, B-cell maturation antigen; GPRC5D, G protein–coupled receptor, class C group 5 member D; FcHR5, fragment crystallizable receptor homolog 5; Rx, treatment lines; ORR, overall response rate; PFS, progression-free survival; DOR, duration of response; ADC, antibody dependent cytotoxicity; FDA, Food and Drug Administration.

*For 200 mg dose.

A real-world evidence study reported the clinical outcomes of 45 patients treated with a selinexor regimen and subsequently received idecabtagene vicleucel, ciltacabtagene autoleucel, or another anti-BCMA CAR-T under development. The majority (75.5%) of patients received low dose selinexor (median 80mg weekly starting dose), most commonly in combination with carfilzomib (35%). The median line of selinexor exposure was seventh, while CAR-T was the ninth. An exploratory multivariate analysis determined that receiving selinexor in the line of therapy prior to CAR-T was associated with the best PFS and OS outcomes with hazard ratios of 0.4 (95% CI 0.113 – 1.09) and 0.08 (95% CI 0.02 – 0.46), respectively (42).

A report of S-containing regimens in RRMM patients who had progressed or failed anti-BCMA CAR-T showed objective responses in 6 of 7 patients, with 1 stringent complete response (treated with SKd), 3 VGPRs (2 treated with SKd and 1 with SVd), and 2 partial responses (PRs, 1 each with Sd and SVd). The ORR was 86% with a clinical benefit rate (CBR) of 100% with 1 additional minor response with SKd (44). A subsequent case series of 11 patients with progression after anti-BCMA (non-CAR-T) treatment (7 were anti-BCMA antibody drug conjugate pretreated) from the STOMP trial, all with weekly dosing of selinexor, reported ORR and CBR rates of 63.6% and 81.8%, respectively, and no cases of disease progression as a best response (23). Median DOR and PFS were not reached after median follow-up of 14.3 months and median OS was 14.8 months (**Table 2**). Response was >6 months for 5/7 responders and up to at least 15.6 months, with 6-month PFS probability determined to be 75%.

## 4 Discussion

Once-weekly SVd, SPd, SDd, and SKd, are all recommended regimen options according to the NCCN^®^ guidelines. Lower starting doses and dose reductions during therapy have shown improved tolerability compared to twice-weekly regimens, while maintaining efficacy—even in difficult-to-treat populations such as TCR or BCMA-refractory MM. We have reviewed clinical trial data supporting the use of lower dose weekly selinexor to maintain efficacy while lowering toxicity. Whether a lower starting dose of selinexor is more effective than higher doses remains to be tested in prospective randomized trials or pharmacokinetic simulation models.

In the BOSTON trial, patients who had a dose reduction had longer PFS, DOR, and time-to-next-treatment, reduced rates of AEs, and improved QoL, suggesting that a strategic dose reduction could optimize selinexor treatment in patients with RRMM (18). Similarly, a retrospective observational study showed that patients with RRMM receiving selinexor-based regimens administered with a lower starting dose and antiemetic support had reduced treatment failure, longer treatment duration, and fewer dose-related toxicities (45). In the STOMP study, lower-dose selinexor maintained anti-MM activity with manageable toxicity, even in heavily pretreated patients.

Spearheaded by anti-CD38 mAbs and the anticipated transition of novel therapies including BCMA targeting CAR-T and bispecifics into earlier treatment lines, patients may be TCR after only a few lines of treatment. Additionally, anti-BCMA refractory MM represents an emerging niche where novel mechanisms of action may provide clinical value. Phase 3 trials enriched for these patients are needed to gain appropriate insights into the sequencing of therapies. The EMN29 study (NCT05028348) is evaluating the SPd-40 triplet in TCE MM with progression of disease after an anti-CD38 antibody in the immediate prior line of treatment. Given that both selinexor and a cereblon E3 ligase modulator such as mezigdomide impact T-cell fitness and support T-cell health) an additional arm of the STOMP trial is underway to evaluate the all-oral triplet combination of selinexor, mezigdomide, and dexamethasone in patients with RRMM who have progressed after either bispecific or CAR-T therapy.

## 5 Conclusions

Most anti-tumor therapies undergo an evolution as experience grows in dosing and schedule - Selinexor is no exception from this posology refinement. S-containing triplets with weekly administration schedules delivered have showed reduced toxicity profiles, improved tolerance and durability of disease response in difficult to manage patient populations. As such, they represent a proven alternative and/or companion class of agent to PIs, IMiDs, and anti-CD38 monoclonal antibodies in heavily pretreated MM. Anti-tumor activity is observed even at low doses of weekly S-containing triplets, where the toxicities of a combination therapy do not exacerbate the AEs expected with the partner drug in a combination. Further research in strategic therapeutic sequencing of selinexor in rational combinations with other classes of anti-MM agents with a focus on optimizing T-cell fitness may facilitate our ability to achieve even better outcomes in multi-class RRMM, which remains area of unmet need.
